# Recent advance in treatment of osteoarthritis by bioactive components from herbal medicine

**DOI:** 10.1186/s13020-020-00363-5

**Published:** 2020-08-01

**Authors:** Xu-zhao Li, Shuai-nan Zhang

**Affiliations:** College of Pharmacy, Guizhou University of Traditional Chinese Medicine, Dong Qing Nan Road, Guian New Area, 550025 People’s Republic of China

**Keywords:** Herbal medicine, Osteoarthritis, Bioactive components, Drug discovery, Drug-likeness properties, Pharmacokinetics

## Abstract

Osteoarthritis (OA) is a common chronic articular degenerative disease, and characterized by articular cartilage degradation, synovial inflammation/immunity, and subchondral bone lesion, etc. The disease affects 2–6% of the population around the world, and its prevalence rises with age and exceeds 40% in people over 70. Recently, increasing interest has been devoted to the treatment or prevention of OA by herbal medicines. In this paper, the herbal compounds with anti-OA activities were reviewed, and the cheminformatics tools were used to predict their drug-likeness properties and pharmacokinetic parameters. A total of 43 herbal compounds were analyzed, which mainly target the damaged joints (e.g. cartilage, subchondral bone, and synovium, etc.) and circulatory system to improve the pathogenesis of OA. Through cheminformatics analysis, over half of these compounds have good drug-likeness properties, and the pharmacokinetic behavior of these components still needs to be further optimized, which is conducive to the enhancement in their drug-likeness properties. Most of the compounds can be an alternative and valuable source for anti-OA drug discovery, which may be worthy of further investigation and development.

## Background

Osteoarthritis (OA) is a common chronic articular degenerative disease, and characterized by articular cartilage degradation, synovial inflammation/immunity, and subchondral bone lesion, etc. [1, 2] The disease affects 2–6% of the population around the world, and its prevalence rises with age and exceeds 40% in people over 70 [1]. Treatment for OA can be divided into non-surgical (e.g. acetaminophen, nonsteroidal anti-inflammatory drugs, and hyaluronic acid, etc.) and surgical (e.g. osteotomy, unicompartmental knee arthroplasty, and total knee arthroplasty) management [1]. However, these current treatments are also accompanied by a series of complications, such as pain, infection, blood problem, and so on [1]. Thus, it can be seen that exploring more safe and effective treatments for OA still need to be carried out on an ongoing basis.

The smooth progress of drug research and development needs the support of the corresponding pathological models. The commonly used methods of mimicking OA include surgical (e.g. Hulth technique, joint immobilization, and destabilization of the medial meniscus, etc.) and non-surgical (e.g. monosodium iodoacetate, papain, and collagenase, etc.) induction [3–8]. The model animals (e.g. mouse, rat, and rabbit, etc.) and human biological samples (e.g. cartilage, peripheral blood mononuclear cell, and fibroblast-like synoviocytes, etc.) are selected as the research object to evaluate the anti-OA mechanism of the drug.

In recent years, increasing interest has been devoted to the treatment or prevention of OA by herbal medicines. OA is a kind of “deficiency syndrome” in traditional Chinese medicine theory [9]. A variety of traditional Chinese medicines with tonifying deficiency effects show the potentials to treat OA [10, 11]. Additionally, herbal compounds with cartilage-protective, anti-inflammatory or antioxidant effects have also been widely used in the treatment of OA [12–14]. Therefore, the herbal compounds with anti-OA activities were reviewed in this paper, and the cheminformatics tools were used to predict their drug-likeness properties and pharmacokinetic parameters, so as to provide the references for their follow-up researches and developments.

## The anti-OA activities of bioactive components from herbal medicines

**Fig. 1:**
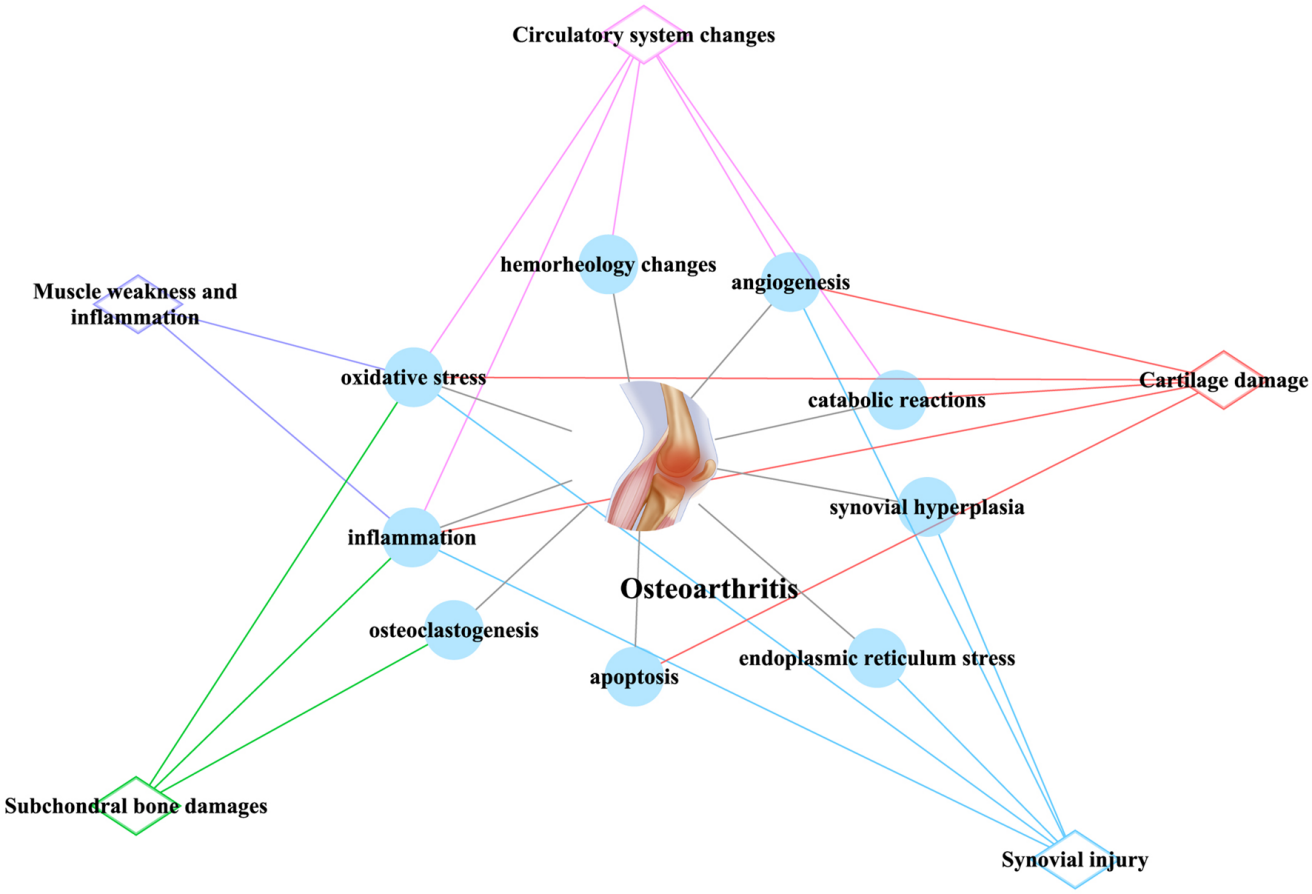
The main pathogenesis and lesion sites of OA. Blue circular node: pathogenesis; red diamond node: cartilage damage; green diamond node: subchondral bone damage; blue diamond node: synovial injury; pink diamond node: circulatory system changes; purple diamond node: muscle weakness and inflammation

**Fig. 2:**
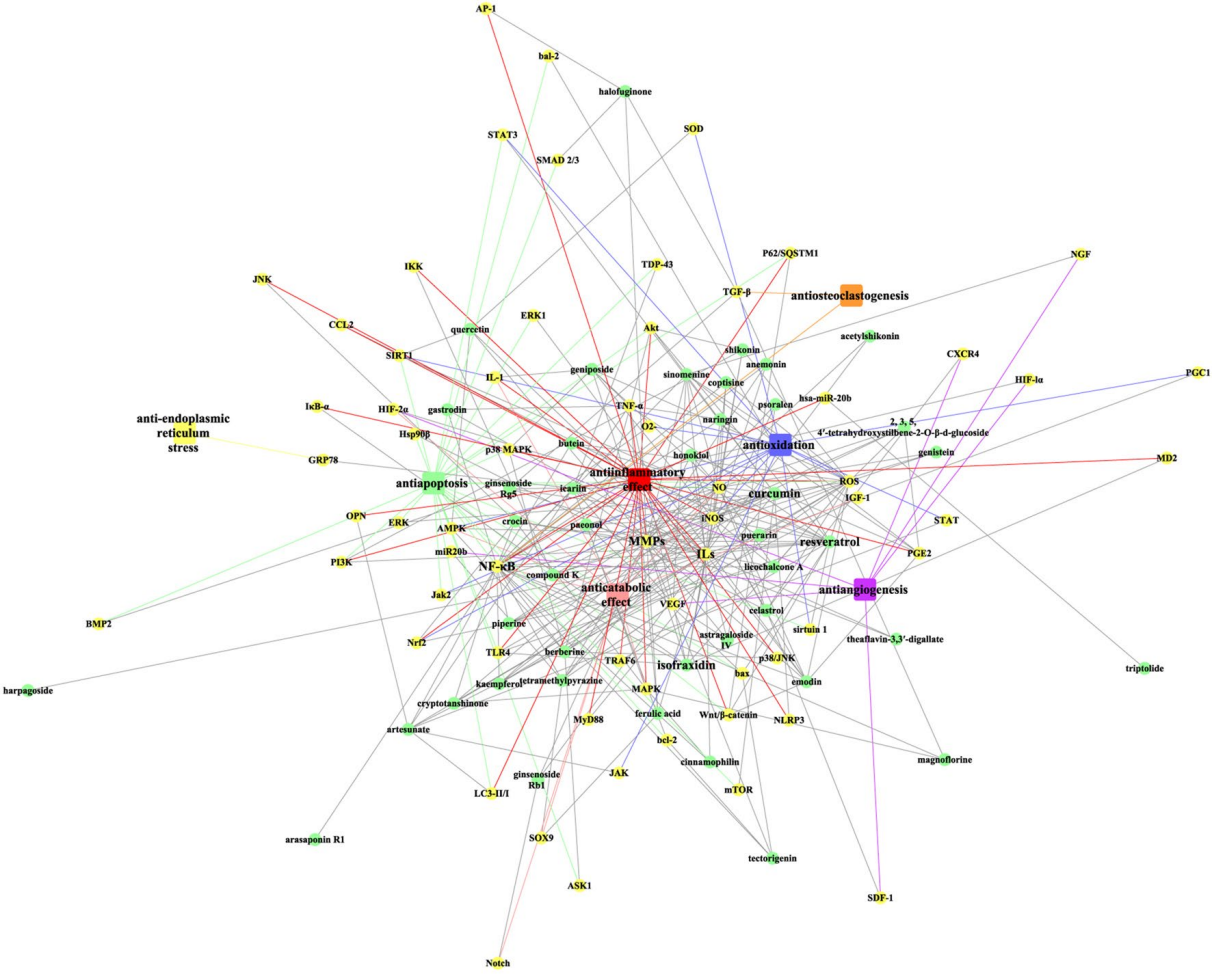
The overview of targets and effects of bioactive components with anti-OA activities. Green circular node: herbal compound; yellow circular node: target; red rectangle node: antiinflammatory effect; green rectangle node: antiapoptosis; blue rectangle node: antioxidation; pink rectangle node: anticatabolic effect; purple rectangle node: antiangiogenesis; orange rectangle node: antiosteoclastogenesis; yellow rectangle node: anti-endoplasmic reticulum stress

**Fig. 3:**
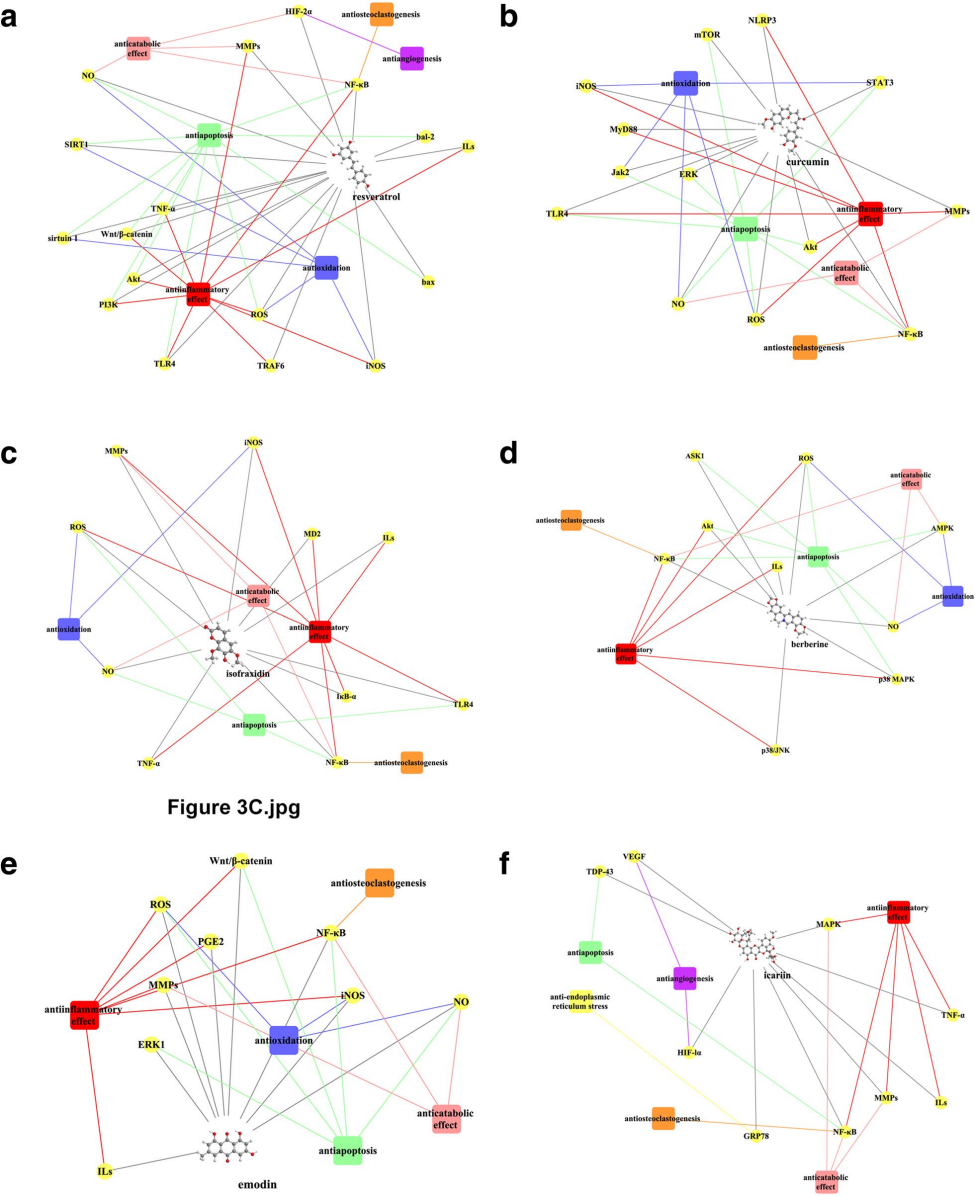
The overview of anti-OA activities of **a** resveratrol, **b** curcumin, **c** isofraxidin, **d** berberine, **e** emodin, and **f** icariin. Yellow circular node: target; red rectangle node: antiinflammatory effect; green rectangle node: antiapoptosis; blue rectangle node: antioxidation; pink rectangle node: anticatabolic effect; purple rectangle node: antiangiogenesis; orange rectangle node: antiosteoclastogenesis; yellow rectangle node: anti-endoplasmic reticulum stress

Information on the treatment of OA by bioactive components from herbal medicines was collected by using Google Scholar (http://scholar.google.com) and PubMed (https://www.ncbi.nlm.nih.gov/pubmed). From herbal medicines, 43 bioactive components with anti-OA activities have been isolated, including 11 terpenoids, 10 flavonoids, 7 alkaloids, 6 phenols, 3 quinones, 2 coumarins, 2 lignans, 1 steroids, and 1 furans (Additional file 1: Figure S1). The networks of OA pathogenesis and compound targets were constructed by Cytoscape software (version 3.8.0). OA is mainly characterized by joint degeneration, meanwhile accompanied by the changes of the related indicators in circulatory system (Fig. 1). Multiple pathological processes are involved in the pathogenesis of OA, such as inflammation, apoptosis, and oxidative stress, etc. (Figure 1). These bioactive components (such as resveratrol, curcumin, and isofraxidin, etc.) mainly target the damaged joints (e.g. cartilage, subchondral bone, and synovium, etc.) and circulatory system to improve the pathogenesis of OA, which mainly exert anti-inflammatory, anti-apoptotic, and anti-oxidative stress effects through interleukin (IL), nuclear factor-κB (NF-κB), and matrix metalloproteinase (MMP) pathways (Figs. 2 and 3). The effective doses of these compounds for the experiment are shown in Table 1.

**Table 1:** The effective doses for the experiment on anti-OA activities of the herbal compounds

No.	Herbal compounds	In vivo	in Vitro
1	2,3,5,4′-Tetrahydroxystilbene-2-O-β-d-glucoside	10–50 mg/kg (rat) [60]	10–400 μg/ml (chondrocyte) [60]
2	Acetylshikonin	5 mg/kg (rat) [86]	3 μM (chondrocyte) [86]
3	Anemonin	2 mg/kg (mouse) [49]	10 μM (chondrocyte) [49]
			10 μM (cartilage explant) [49]
4	Arasaponin R1		125 mg/l (chondrocyte) [41]
5	Artesunate	25–300 mg/kg (rat) [57, 100]	
6	Astragaloside IV		25–500 mmol/l (chondrocyte) [52, 81, 90]
			50 µg/ml (chondrocyte) [73]
7	Berberine	10–50 mg/kg (rat) [17]	20–100 μM (OA synovial fibroblasts) [17]
		10––200 μM/50 μl (rat) [67, 68]	25–100 μM (chondrocyte) [67–69]
		7–28 μg/kg (rat) [69]	25–100 μM (cartilage explant) [68]
8	Butein	20 mg/kg (mouse) [46]	10–50 μM (chondrocyte) [46]
9	Celastrol	1 mg/kg (rat) [84]	0.1–1 μM (chondrocyte) [54]
10	Cinnamophilin		5–30 μM (SW1353 cell) [30]
11	Compound K		0.01–10 μM (MC3T3-E1 cell) [37]
12	Coptisine		2.5-10 μg/ml (chondrocyte) [44]
13	Crocin	5–100 μM/0.3 ml (rabbit) [43]	50–100 μM (chondrocyte) [43]
		30 mg/kg (rat) [110]	
14	Cryptotanshinone	10 mg/kg (mouse) [29]	5–20 μM (chondrocyte) [29]
15	Curcumin	50 μM (mouse) [15]	10 μM (THP-1 cell) [15]
		50–100 mg/kg (mouse) [27, 65, 66]	50–100 μM (chondrocyte) [27, 28]
		200 mg/kg (rat) [96]	40 μM (synoviocyte) [97]
16	Emodin	5–25 μM/50 µl (rat) [39]	5–30 µg/ml (chondrocyte) [38, 39]
17	Ferulic acid		40 μM (chondrocyte) [55]
18	Gastrodin	100 μg/kg (rat) [51]	10–50 μM (chondrocyte) [51]
19	Geniposide	40 mg/kg (rabbit) [31]	80 μg/ml (chondrocyte) [31]
20	Genistein	0.3–0.5 mg/kg (rabbit) [58]	
21	Ginsenoside Rb1	80 μM/0.3 ml (rat) [76]	20–80 μM (SW1353 cell) [76]
		300 μM/200 μl (rat) [87]	
22	Ginsenoside Rg5	10–15 mg/kg (rat) [74]	
23	Halofuginone	0.2–2.5 mg/kg (mouse) [20, 82, 91]	
24	Harpagoside		300 μg/ml (chondrocyte) [32]
25	Honokiol		2.5–10 μM (chondrocyte) [50]
26	Icariin	10–40 ng/ml (rat) [9]	20 μM (SW1353 cell) [16, 79]
		20 μM (rat) [16]	12.5 mg/l (chondrocyte) [41]
		1–6 g/kg (rabbit) [40]	12 μg/ml (SW1353 cell) [92]
		10–25 mg/kg (mouse) [78, 108]	0.5–1 μM (OA fibroblast-like synoviocyte) [95]
27	Isofraxidin	20 mg/kg (mouse) [34]	1–50 μM (chondrocyte) [33, 34]
28	Kaempferol		25–100 µM (chondrocyte) [47]
29	Licochalcone A		5–20 µM (chondrocyte) [36]
30	Magnoflorine	50 ng/2 μl (pig) [23]	25 μg/ml (MC3T3-E1 cell) [23]
31	Naringin	100 mg/kg (mouse) [42]	5 µM (chondrocyte) [42]
		5–10 mg/kg (rat) [106]	
32	Paeonol	20 mg/kg (rat) [56]	50 µM (chondrocyte) [56]
		0.2–0.8 mg/kg (rabbit) [71]	
33	Piperine		10–100 μg/ml (chondrocyte) [45]
34	Psoralen	1 mg/kg (rat) [22]	10 µM (chondrocyte) [22]
			10 µM (synoviocyte) [22]
			10^−6^ mol/l (chondrocyte) [75]
35	Puerarin	25–50 mg/kg (mouse) [53]	50 nM (chondrocyte) [53]
		30–200 mg/kg (rat) [83, 88]	
36	Quercetin	50–100 mg/kg (rat) [21, 84]	25 μM (chondrocyte) [21]
		25 mg/kg (rabbit) [98]	
37	Resveratrol	45 mg/kg (mouse) [12, 25]	50 μM (SW1353 cell) [12]
		10–50 μM/kg (rabbit) [14, 61]	10–100 μM (chondrocyte) [24, 26, 62]
		30–120 mg/kg (rabbit) [64, 109]	1–5 μM (peripheral blood mononuclear cell) [63]
		10–100 μg/8 μl (mouse) [77]	
38	Shikonin	10 mg/kg (rat) [35]	50 μM (chondrocyte) [86]
39	Sinomenine	2 mg/kg (rabbit) [99]	10–250 μM (chondrocyte) [72]
		5 mg/0.2 ml (rabbit) [102]	10–250 μM (cartilage explant) [72]
			0.25 mM (mesenchymal stem cell) [93]
40	Tectorigenin	0.75–1.5 μg/kg (rat) [48]	50–100 μM (chondrocyte) [48]
41	Tetramethylpyrazine	30–100 mg/kg (rat) [19, 89]	0.5–200 μM (chondrocyte) [13, 70]
		2.1 mg/0.1 ml (rat) [80]	50–200 μM (cartilage explant) [70]
42	Theaflavin-3,3′-digallate		25–75 μg/ml (chondrocyte) [59]
43	Triptolide	0.35 μg (mouse) [107]	20 ng/ml (THP-1 cell) [107]

### The effects of bioactive components on cartilage in OA

Cartilage is pivotal to the normal function of synovial joints. Cartilage covers and protects the ends of long bones permitting friction-free locomotion and movement at the joints. A dysfunction in the cartilage is one of the important inducing factors and pathological features of OA [14]. Cartilage consists of chondrocytes that generate a large of collagenous extracellular matrix, proteoglycans, and elastin fibers. Histological analysis shows that various components can repair the damage of chondrocytes in OA, including resveratrol [14], curcumin [15], icariin [16], berberine [17], sinomenine [18], tetramethylpyrazine [19], halofuginone [20], quercetin [21], psoralen [22], and magnoflorine [23].

The inflammatory mediators lead to articular cartilage damage and the clinical manifestations of OA [24]. Resveratrol attenuates inflammation through NF-κB, toll-like receptor 4 (TLR4)/tumor necrosis factor receptor–associated factor 6 (TRAF6), and Wnt/β-catenin signaling pathways [12, 24–26]. Curcumin reduces the expression of pro-inflammatory mediators via inhibiting the activation of NLR pyrin domain containing 3 inflammasome and NF-κB [15, 27, 28]. Cryptotanshinone [29] and cinnamophilin [30] inhibit IL-1β-induced cartilage inflammation through suppressing NF-κB and mitogen-activated protein kinase (MAPK) activation. Geniposide may have anti-inflammatory potential on OA, and p38 MAPK signaling is a crucial pathway for this effect [31]. Harpagoside exerts anti‐inflammatory effect via suppressing c‐fos/activator protein‐1 activity in OA chondrocytes [32]. Isofraxidin targets the TLR4/myeloid differentiation protein-2 axis and NF-κB signaling pathway to prevent OA inflammation [33, 34]. Shikonin inhibits chondrocyte inflammation by the regulation of the phosphatidylinositol-3-kinase (PI3K)/Akt signaling pathway in OA rats [35]. Anti-inflammatory effects of licochalcone A are associated with NF-κB and nuclear factor (erythroid-derived 2)-like 2 signaling pathways [36]. Compound K, an IkBα kinase inhibitor, may alleviate inflammatory response in cartilage [37]. Emodin ameliorates OA cartilage inflammation by inhibiting NF-κB and Wnt/β-catenin signaling [38, 39]. NF-κB signaling pathway is also involved in the treatment of cartilage inflammation by icariin [40, 41], arasaponin R1 [41], berberine [17], tetramethylpyrazine [13], naringin [42], crocin [43], coptisine [44]; piperine [45], butein [46]; kaempferol [47], tectorigenin [48], anemonin [49], honokiol [50], and gastrodin [51]. In addition, some molecules have been reported to reduce the expression of inflammatory factors in OA cartilage, but the related pathways still need to be further explored, such as astragaloside IV [52], puerarin [53], celastrol [54], ferulic acid [55], paeonol [56], artesunate [57], genistein [58], theaflavin-3,3′-digallate [59], and 2, 3, 5,4′-tetrahydroxystilbene-2-O-β-d-glucoside [60].

In the progressive stage of OA, apoptosis destroys chondrocyte homeostasis [61]. Resveratrol inhibits chondrocyte apoptosis in OA through a variety of signaling pathways, including nitric oxide (NO) [61], NF-κB [26], sirtuin 1 [62, 63], Wnt/β‑catenin [62], bal-2/bax [64], TLR4 [12], and PI3K/Akt signaling pathways [12]. Curcumin reverses apoptosis of chondrocytes via modulating the balance of antiapoptotic and proapoptotic proteins [15]. This is related to janus kinase 2/signal transducer and activator of transcription 3 [65], extracellular signaling-regulated kinase (ERK) 1/2, and Akt/mammalian target of rapamycin (mTOR) pathways [66]. Berberine prevents NO-induced chondrocyte apoptosis via AMP‐activated protein kinase (AMPK) and p38 MAPK signaling [67, 68], and promotes cell survival through activating Akt signaling in OA model [69]. Tetramethylpyrazine inhibits the chondrocytes apoptosis through suppressing the production of reactive oxygen species (ROS) [70] and inactivating NF-κB signaling pathway [13]. Paeonol alleviates chondrocyte apoptosis by regulating the levels of ROS, bcl-2, and bax [56, 71]. Some components (icariin [9], sinomenine [72], astragaloside IV [73], quercetin [21], shikonin [35], tectorigenin [48], gastrodin [51], and ginsenoside Rg5 [74]) also exert anti-apoptotic effects on chondrocytes through various mechanisms. The promoting effects of puerarin [53], psoralen [75], magnoflorine [23], and emodin [38] on proliferation may be also beneficial to reverse cartilage apoptosis.

The extracellular matrix of articular cartilage is mainly composed of type II collagen and aggrecan. Catabolic reactions take place in the OA cartilage, in which collagen and aggrecan are degraded [63]. MMPs are a family of zinc containing, calcium-dependent neutral proteases which can initiate the cleavage of type II collagen and aggrecan [76]. In OA chondrocytes, resveratrol may reverse the decrease in the levels of type II collagen, aggrecan, and glycosaminoglycan by regulating silent information regulator 2 type 1, hypoxia-inducible factor-2α, and MMPs expression [24, 62, 63, 77]. Curcumin [28], naringin [42], icariin [16, 78, 79], berberine [68, 69], sinomenine [72], tetramethylpyrazine [13, 70, 80], astragaloside IV [81], halofuginone [82], puerarin [83], quercetin [84], celastrol [54, 85], harpagoside [32], ferulic acid [55], shikonin, acetylshikonin [86], ginsenoside Rb1 [76, 87], cinnamophilin [30], honokiol [50], 2, 3, 5, 4′-tetrahydroxystilbene-2-O-β-d-glucoside [60], geniposide [31], ginsenoside Rg5 [74], cryptotanshinone [29], isofraxidin [33], paeonol [56], crocin [43], coptisine [44], piperine [45], butein [46], licochalcone A [36], tectorigenin [48], theaflavin-3,3′-digallate [59], anemonin [49], gastrodin [51], compound K [37], and emodin [38, 39] inhibit the expression of MMPs through a variety of pathways, such as IL-1β signaling, NF-κB signaling, AMPK signaling, MAPK signaling, and NO signaling, etc. The inhibition of cartilage catabolic processes by resveratrol [24], curcumin [66], and astragaloside IV [73] may be also related to their regulation on autophagy, activation of which may reduce the severity of OA. Additionally, Artesunate [57] and psoralen [75] can markedly enhance the expression of type II collagen as well.

Oxidative stress plays a crucial role in the progression of OA, and the dysregulation of various oxidative stress indices occurs in cartilage, such as NO, inducible NO synthase (iNOS), and ROS, etc. [54]. Resveratrol [61], tetramethylpyrazine [13, 70], celastrol [54], isofraxidin [33, 34], paeonol [56], shikonin [35], coptisine [44], piperine [45], butein [46], genistein [58], kaempferol [47], licochalcone A [36], honokiol [50], 2, 3, 5, 4′-tetrahydroxystilbene-2-O-β-d-glucoside [60], compound K [37], geniposide [31], emodin [38], and curcumin [65] may reverse the abnormal expression of these indexes. Mitochondrial dysfunction in chondrocytes is associated with OA, and induces oxidative stress [88]. Puerarin [88] and quercetin [84] may attenuate mitochondrial dysfunction in OA rats. Subsequently, oxidative stress induces endoplasmic reticulum stress in OA, and quercetin may also repress this process by activating the sirtuin1/AMPK signaling pathway [21].

Abnormal angiogenesis is also closely related to the development of OA [57]. Some herbal compounds (e.g. sinomenine [18], tetramethylpyrazine [89], astragaloside IV [90], and artesunate [57]) may suppress aberrant angiogenesis by interfering with a variety of targets, such as vascular endothelial growth factor (VEGF), miR20b, and nerve growth factor (NGF), etc.

### The effects of bioactive components on subchondral bone in OA

Besides cartilage, subchondral bone lesions are the characteristic pathological changes in OA as well [91]. The micro-computed tomography scan shows that halofuginone restores coupled bone remodelling and aberrant angiogenesis in subchondral bone [82, 91]. Osteoclast is a type of bone cell breaking down bone tissue, and collagen degradation mediated by which is also involved in the pathophysiology of OA [57]. Icariin [92] and sinomenine [93] suppress osteoclastogenesis through osteoprotegerin-NF-κB system. Halofuginone suppresses Th17-induced osteoclastogenesis via inhibition of TGF-β signaling [82]. Artesunate interrupts anterior cruciate ligament transection-associated osteoclastogenesis [57]. In addition to osteoclasts, osteoblasts are also the major cellular component of bone, which synthesize dense and crosslinked collagen and reshape bone tissue. Magnoflorine [23] and compound K [37] stimulate osteoblast proliferation, differentiation, and mineralization. Resveratrol may play the roles on alkaline phosphatase activity, osteocalcin release, and mineralization in osteoblasts via promoting the Wnt/β-catenin signaling pathway [94]. Histological analysis indicates that cryptotanshinone [29], isofraxidin [34], and resveratrol [77] may reduce subchondral bone plate thickness.

### The effects of bioactive components on synovium in OA

Synovium supplies nutrients to cartilage and protects the joint structures and the adjoining musculoskeletal tissues [95]. OA is a classic degenerative synovial disease. Synovitis affects both symptoms and progression of OA [95]. Curcumin [96, 97], icariin [95], psoralen [22], berberine [17], quercetin [98], geniposide [31], sinomenine [99], and artesunate [57, 100] produce anti-inflammatory activity in synoviocytes/synovia by regulating the levels of various inflammatory factors, such as MMPs, ILs, and tumor necrosis factor (TNF)-α, etc. Synovial proliferation is induced by inflammation in OA [101]. The antiproliferative effects of curcumin [97] and icariin [95] may reverse this process. Likewise, angiogenesis and inflammation are closely associated in OA [57]. Sinomenine [18] and artesunate [57] may prevent the expression of angiogenic factors (e.g. VEGF, NGF, and angiopoietin-1, etc.). Oxidative stress and inflammation promote each other in joints [98]. Quercetin [98] and geniposide [31] may inhibit oxidative stress in synovial region. Glucose-regulated protein-78 aggregates in the endoplasmic reticulum, which is widely used as a marker for endoplasmic reticulum stress. Icariin can reduce Glucose-regulated protein-78 expression in synovium of OA [95]. Geniposide [31] and sinomenine [102] can decrease the levels of MMPs and cartilage oligomeric matrix proteins in synovial fluid, which may help to alleviate the process of cartilage degradation in OA. Insulin-like growth factor-1 accelerates the differentiation of chondrocytes, stimulates the synthesis of cartilage matrix, and inhibits the matrix decomposition, the up-regulation of which by artesunate may facilitate cartilage protection in OA [100].

### The effects of bioactive components on circulatory system in OA

Circulatory pathology is closely related with OA [103]. A variety of herbal compounds can reverse some pathological processes in serum of OA model. Quercetin [104], resveratrol [63, 105], sinomenine [99], puerarin [53], isofraxidin [34], naringin [106], ginsenoside Rb1 [87], triptolide [107], and icariin [108] can reduce the serum levels of inflammatory cytokines, such as ILs, TNF-α, and hsa-miR-20b, etc. Sinomenine [102], quercetin [98, 104], and artesunate [100] may regulate the expressions of cartilage catabolic factors (e.g. MMPs, tissue inhibitors of MMP, and a disintegrin and metalloproteinase with thrombospondin motifs, etc.) in serum. Icariin reduces VEGF and hypoxia-inducible factor‐lα levels in the peripheral blood, which may help to inhibit the formation of new blood vessels in the synovial tissue of joints [9]. Resveratrol effectively improves the blood rheology, which facilitates to prevent and delay the degenerative changes in the articular cartilage of OA model [109]. Additionally, quercetin increases serum superoxide dismutase level, which is a major active molecule to scavenge free radical [98].

### The effects of bioactive components on muscle in OA

Muscle weakness and inflammation also play a role in OA development and progression [110]. Crocin attenuates OA symptoms through alleviating muscle oxidative stress (targets: nuclear factor (erythroid-derived 2)-like 2, superoxide anion, and glutathione, etc.) and inflammation (pathways: c-Jun N-terminal kinase, NF-κB, and MAPK, etc. signaling pathways) induction [110].

## Pharmacokinetic parameters and drug-likeness properties prediction of bioactive components with anti-OA activities

In addition to their therapeutic activities, the pharmacokinetic behaviors of these components are also the key factors affecting their ability to develop drugs. Only the compounds with good drug-likeness properties have the possibility to be further investigated and developed. Thus, in the following section, the cheminformatics tools were applied to predict the pharmacokinetic parameters and drug-likeness properties of these compounds.

The pharmacokinetic parameters of these compounds were calculated by using pkCSM (http://biosig.unimelb.edu.au/pkcsm/prediction) [111]. The compounds depicted as 2D structures in the MDL Molfile format were imported into the website. The water solubility of the compounds can influence their efficacy in vivo. The good aqueous solubility can facilitate the molecules dispersing into biological body fluids, thereby expediting their absorption and distribution processes [112]. Water solubility assessment showed that most of herbal compounds (38/43) were soluble in water (> − 4 log mol/L), while five of 43 molecules were low soluble in water (< − 4 log mol/L, Table 2). The low solubility of curcumin is one of the factors affecting its oral bioavailability. Reportedly, the solubility of curcumin can be significantly improved by addition of an electron-withdrawing group. A chemically modified curcumin, TRB-n0224, also has good therapeutic effects on OA model [113].

**Table 2:** Pharmacokinetic parameters and drug-likeness properties prediction of herbal compounds with anti-OA activities

No.	Herbal compounds	Botanical source	Water solubility (log mol/L)	Skin permeability (log Kp)	VDss (human) (log L/kg)	Total clearance (log ml/min/kg)	DL
1	2, 3, 5, 4′-Tetrahydroxystilbene-2-*O*-β-d-glucoside	*Polygonum multiflorum* Thunb.	− 3.227	− 2.735	− 0.109	0.219	0.16
2	Acetylshikonin	*Lithospermum erythrorhizon* Sieb.	− 3.022	− 3.188	0.084	0.336	0.41
3	Anemonin	*Clematis* L.	− 1.436	− 3.646	− 0.005	0.431	− 1.57
4	Arasaponin R1	*Panax notoginseng* (Burkill) F. H. Chen	− 2.765	− 2.735	− 0.239	0.497	0.24
5	Artesunate	*Artemisia annua* L.	− 3.125	− 2.734	0.286	0.973	− 0.39
6	Astragaloside IV	*Astragalus membranaceus* (Fisch.) Bge.	− 2.693	− 2.735	− 0.507	0.147	0.05
7	Berberine	*Hydrastis canadensis* L., *Phellodendron amurense* Rupr., and *Coptis chinensis* Franch.	− 3.341	− 2.734	0.764	1.272	0.91
8	Butein	*Rhus verniciflua* Stokes	− 2.857	− 2.835	0.003	0.062	0.82
9	Celastrol	*Celastrus aculeatus* Merr.	− 4.584	− 2.720	− 0.987	− 0.090	0.63
10	Cinnamophilin	*Cinnamomum philippinense* (Merr.) C. E. Chang	− 4.465	− 3.051	0.195	0.215	0.76
11	Compound K	*Panax ginseng* C. A. Mey.	− 3.683	− 2.735	− 0.627	0.475	0.34
12	Coptisine	*Coptis chinensis* Franch.	− 3.325	− 2.734	0.636	1.298	− 0.08
13	Crocin	*Crocus sativus* L.	− 2.804	− 2.735	− 0.294	1.768	− 0.27
14	Cryptotanshinone	*Salvia miltiorrhiza* Bunge	− 4.571	− 2.563	0.689	0.841	0
15	Curcumin	*Curcuma longa* L.	− 4.926	− 2.913	− 0.184	0.033	− 0.66
16	Emodin	*Rheum palmatum* L.	− 3.179	− 2.764	0.045	0.348	− 0.72
17	Ferulic acid	*Oldenlandia diffusa* (Willd.) Roxb.	− 1.737	− 2.621	− 0.642	0.653	− 0.44
18	Gastrodin	*Gastrodia elata* Blume	− 1.354	− 2.985	− 0.463	0.234	− 1.19
19	Geniposide	*Gardenia jasminoides* J. Ellis	− 2.534	− 2.914	− 0.415	1.408	0.51
20	Genistein	*Glycine max* (Linn.) Merr.	− 3.533	− 2.737	− 0.709	0.232	0.71
21	Ginsenoside Rb1	*Panax ginseng* C. A. Mey.	− 2.839	− 2.735	− 0.440	0.570	0.28
22	Ginsenoside Rg5	*Panax ginseng* C. A. Mey.	− 3.520	− 2.735	− 1.033	0.513	0.44
23	Halofuginone	*Dichroa febrifuga* Lour.	− 3.613	− 2.960	0.593	1.134	0.91
24	Harpagoside	*Harpagophytum procumbens* DC.	− 3.181	− 2.751	− 0.332	1.057	− 0.96
25	Honokiol	*Magnolia officinalis* Rehd. et Wils.	− 3.862	− 2.795	0.350	0.377	− 0.33
26	Icariin	*Epimedium brevicornu* Maxim.	− 2.930	− 2.735	− 0.278	0.076	1.09
27	Isofraxidin	*Acanthopanax senticosus* (Rupr. & Maxim.) Harms	− 2.37	− 2.728	− 0.382	0.762	− 0.88
28	Kaempferol	*Kaempferia rotunda* L.	− 3.176	− 2.735	− 0.107	0.558	0.77
29	Licochalcone A	*Glycyrrhiza uralensis* Fisch.	− 4.161	− 2.808	0.092	0.482	− 0.16
30	Magnoflorine	*Phellodendron chinense* Schneid.	− 3.447	− 2.954	1.306	1.102	0.8
31	Naringin	Citrus plants	− 3.103	− 2.735	0.157	0.685	1.21
32	Paeonol	*Paeonia suffruticosa* Andr.	− 1.606	− 2.758	0.137	0.630	0.01
33	Piperine	*Piper nigrum* L.	− 3.799	− 2.824	0.266	0.240	− 0.02
34	Psoralen	*Psoralea corylifolia* L.	− 2.688	− 2.271	− 0.284	0.738	− 0.93
35	Puerarin	*Pueraria lobata* (Willd.) Ohwi	− 3.845	− 2.735	− 0.217	0.183	0.04
36	Quercetin	*Cudrania tricuspidata* (Carr.) Bur.	− 2.942	− 2.735	0.134	0.515	0.93
37	Resveratrol	*Polygonum cuspidatum* Sieb.*, Veratrum album* var. *grandiflorum* Maxim, and *Vitis vinifera* L. etc.	− 3.285	− 3.132	0.118	0.141	− 0.94
38	Shikonin	*Lithospermum erythrorhizon* Sieb.	− 2.535	− 2.775	0.297	0.105	0.36
39	Sinomenine	*Sinomenium acutum* (Thunb.) Rehd. et Wils.	− 2.276	− 3.550	0.673	0.921	0.87
40	Tectorigenin	*Belamcanda chinensis* (L.) Redouté	− 3.580	− 2.737	− 0.644	0.166	0.58
41	Tetramethylpyrazine	*Ligusticum chuanxiong* Hort.	− 0.786	− 2.671	− 0.136	0.551	− 1.53
42	Theaflavin-3,3′-digallate	Black tea	− 2.892	− 2.735	− 0.087	0.242	0.47
43	Triptolide	*Tripterygium wilfordii* Hook.f.	− 3.657	− 3.202	0.465	0.484	− 0.32

Water solubility: the solubility of the molecule in water at 25 °C; less than − 10: insoluble; between − 10 and − 6: poorly soluble; between − 6 and − 4: moderately soluble; between − 4 and − 2: soluble; between − 2 and 0: very soluble; more than 0: highly soluble

Skin permeability: the human skin permeability of compounds in vitro; more than − 2.5: low skin permeability; less than − 2.5: high skin permeability

VDss (human): the volume of compounds distributed in tissue; less than − 0.15: low distribution; more than 0.45: high distribution

Total clearance: a combination of hepatic clearance and renal clearance; less than 0.25: low total clearance; more than 0.25: high total clearance

DL: drug-likeness model score; between 0 and 2: very drug-like molecules; between − 3 and − 0.5: non-drug like molecules

The main pathological features of OA are the degenerative lesions of cartilage and synovium in the joint. It is not conducive to the treatment of local lesions of OA if the distribution of drug molecules in the blood is more than that in the lesion tissues. VDss index can be used to predict the distribution of molecules in tissue and plasma. VDss analysis showed that thirty-six of 43 herbal compounds were mainly distributed in the circulatory system (< 0.45 log L/kg, Table 2). This may require some measures to increase their levels in local tissues. Intra-articular injection allows the molecules to accumulate in the joint cavity, thus enhancing their effects on local lesions. Intra‐articular delivery of resveratrol [77], tetramethylpyrazine [80], and anemonin [49] may enhance their articular cavity retention for treating OA.

In addition to intra-articular injection, transdermal delivery of joint is also one of the local administration methods. Extra-articular percutaneous approach has advantages over intra-articular injection, such as greater safety, easier use, better patient compliance, and so on. Skin permeability is the necessary requirement for transdermal drug delivery. Skin permeability estimation indicated that almost all of these herbal molecules (42/43) were easy to penetrate into the epidermis (prediction value less than − 2.5, Table 2), especially anemonin, sinomenine, and triptolide.

The low clearance rate of drugs results in the prolongation of their half-life in vivo. This may produce a sustained and stable curative effect on the chronic diseases, such as OA. At the same time, however, attention should also be paid to the cumulative dose of herbal components with low total clearance. These compounds may also cause cumulative toxicity when they are used for long-term therapeutic purposes. Total clearance prediction showed that fifteen of 43 herbal compounds have the low hepatic clearance and renal clearance rates (prediction value less than 0.25, Table 2), especially celastrol, curcumin, and butein.

The drug-likeness properties prediction of these herbal compounds was analyzed by using MolSoft online tools (http://molsoft.com/mprop/) [114]. The input for the analysis was the MDL Molfile format of these compounds. Over half of these molecules (26/43) had the great possibility of becoming the drugs (prediction value between 0 and 2, Table 2), which have the possibility of being further studied and developed. However, of these compounds, nine molecules had poor drug-likeness properties (prediction value between -3 and − 0.5, Table 2), which may require some measures to optimize their pharmacokinetics parameters, such as molecular modification, drug administration route change, and drug dosage form optimization, etc.

## Conclusion and future directions

In this review, we have summarized and analyzed 43 herbal compounds with anti-OA activities. The main therapeutic sites of these molecules for the treatment of OA are articular cartilage, subchondral bone, synovial membrane, and circulatory system, etc. Over half of these compounds have good drug-likeness properties (e.g. naringin, icariin, and quercetin, etc.), which may be worthy of further investigation and development. In addition, these compounds are mainly isolated from *Araliaceae, Leguminosae*, and *Polygonaceae* plants, etc., which would get more attention in the following researches.

Through cheminformatics analysis, the pharmacokinetic behavior of these components still needs to be further optimized, which is conducive to the enhancement in their drug-likeness properties. The water solubility of molecules can be changed by mean of structural modification, so as to enhance their oral absorption process. In the subsequent distribution process, the accumulation of drug molecules in the joint tissues is conducive to the treatment of the main lesion sites of OA. Both intra-articular injection and articular percutaneous administration can increase the levels of drug molecules in the joint, between which the latter one has a stronger application potential in the treatment of OA. Additionally, the retention time of the components with low clearance rate is increased in vivo, which is conducive to the continuous treatment of OA. However, when used for a long time, their doses should be properly adjusted to avoid cumulative toxicity.

At present, the application of herbal compounds in the treatment of OA has made some progress. However, compared to other arthritis (such as rheumatoid arthritis (RA)), the application of herbal compounds in OA is still inadequate. There is some common pathogenesis between OA and RA, such as inflammation, apoptosis, and oxidative stress, etc. [112]. Therefore, the potential of anti-RA drugs in the treatment of OA would be further explored in future researches. In addition, some new research patterns can be used to speed up the exploration of the mechanism and chemical basis of herbs in the treatment of OA, such as biolabelled research pattern [115, 116], chinmedomics [117], and systems pharmacology [118], etc.

## Supplementary information

**Additional file 1: Figure S1.** Molecular structures of bioactive components isolated from herbal medicines treating OA.

## Data Availability

Not applicable.

## Abbreviations

AMPK: AMP‐activated protein kinase
ERK: Extracellular signaling-regulated kinase
IL: Interleukin
iNOS: Inducible NO synthase
MAPK: Mitogen-activated protein kinase
MMP: Matrix metalloproteinase
mTOR: Mammalian target of rapamycin
NF-κB: Nuclear factor-κB
NGF: Nerve growth factor
NO: Nitric oxide
OA: Osteoarthritis
PI3K: Phosphatidylinositol-3-kinase
RA: Rheumatoid arthritis
ROS: Reactive oxygen species
TLR4: Toll-like receptor 4
TNF: Tumor necrosis factor
TRAF6: Tumor necrosis factor receptor–associated factor 6
VEGF: Vascular endothelial growth factor

## References

[1] Hsu H, Siwiec RM (2019). Knee osteoarthritis statpearls.

[2] de Lange-Brokaar BJ, Ioan-Facsinay A, van Osch GJ, Zuurmond AM, Schoones J, Toes RE (2012). Synovial inflammation, immune cells and their cytokines in osteoarthritis: a review. Osteoarthritis Cartilage.

[3] Glasson SS, Blanchet TJ, Morris EA (2007). The surgical destabilization of the medial meniscus (DMM) model of osteoarthritis in the 129/SvEv mouse. Osteoarthritis Cartilage.

[4] Combe R, Bramwell S, Field MJ (2004). The monosodium iodoacetate model of osteoarthritis: a model of chronic nociceptive pain in rats?. Neurosci Lett.

[5] Kikuchi T, Sakuta T, Yamaguchi T (1998). Intra-articular injection of collagenase induces experimental osteoarthritis in mature rabbits. Osteoarthritis Cartilage.

[6] Videman T (1982). Experimental osteoarthritis in the rabbit: comparison of different periods of repeated immobilization. Acta Orthop Scand.

[7] Hulth A (1982). Experimental osteoarthritis: a survey. Acta Orthop Scand.

[8] Bentley G (1971). Papain-induced degenerative arthritis of the hip in rabbits. J Bone Joint Surg Br.

[9] Huang H, Zhang ZF, Qin FW, Tang W, Liu DH, Wu PY (2019). Icariin inhibits chondrocyte apoptosis and angiogenesis by regulating the TDP-43 signaling pathway. Mol Genet Genomic Med..

[10] Liang J (2010). Pathology Observation of Acanthopanax Senticosus on Knee Osteoarthritis of Rabbit. J Liaoning Univ TCM..

[11] Shin JS, Park N, Ra J, Kim Y, Shin M, Hong M (2009). Panax ginseng C.A. Meyer modulates the levels of MMP3 in S12 murine articular cartilage cell line. J Ethnopharmacol.

[12] Xu X, Liu X, Yang Y, He J, Gu H, Jiang M, et al. Resveratrol inhibits the development of obesity-related osteoarthritis via the TLR4 and PI3K/Akt signaling pathways. Connect Tissue Res. 2019:1–12. 10.1080/03008207.2019.1601187 30922122

[13] Yu T, Qu J, Wang Y, Jin H (2018). Ligustrazine protects chondrocyte against IL-1beta induced injury by regulation of SOX9/NF-kappaB signaling pathway. J Cell Biochem.

[14] Elmali N, Esenkaya I, Harma A, Ertem K, Turkoz Y, Mizrak B (2005). Effect of resveratrol in experimental osteoarthritis in rabbits. Inflamm Res.

[15] Sun Y, Liu W, Zhang H, Li H, Liu J, Zhang F (2017). Curcumin prevents osteoarthritis by inhibiting the activation of inflammasome NLRP3. J Interferon Cytokine Res.

[16] Zeng L, Wang W, Rong XF, Zhong Y, Jia P, Zhou GQ (2014). Chondroprotective effects and multi-target mechanisms of Icariin in IL-1 beta-induced human SW 1353 chondrosarcoma cells and a rat osteoarthritis model. Int Immunopharmacol.

[17] Liu SC, Lee HP, Hung CY, Tsai CH, Li TM, Tang CH (2015). Berberine attenuates CCN2-induced IL-1beta expression and prevents cartilage degradation in a rat model of osteoarthritis. Toxicol Appl Pharmacol.

[18] Zheng J, Wang R, Kou J, Luo H (2016). Effects of Sinomenine on expressions of VEGF and NGF in articular cartilage and synovium of rabbit knee osteoarthritis models. Chin J Inform TCM..

[19] Xu D, Shen W (2007). Ligustrazine reduces cartilage destruction in model of osteoarthritis in rats. J Med Bull Shanghai Jiaotong U..

[20] Li J, Xu B, Guo W, Mu W, Zhang Z, Ji B (2017). Halofuginone delays articular cartilage degeneration in early osteoarthritis. J Chin J Tissue Eng Res..

[21] Feng K, Chen Z, Pengcheng L, Zhang S, Wang X (2019). Quercetin attenuates oxidative stress-induced apoptosis via SIRT1/AMPK-mediated inhibition of ER stress in rat chondrocytes and prevents the progression of osteoarthritis in a rat model. J Cell Physiol.

[22] Wang C, Al-Ani MK, Sha Y, Chi Q, Dong N, Yang L (2019). Psoralen protects chondrocytes, exhibits anti-inflammatory effects on synoviocytes, and attenuates monosodium iodoacetate-induced osteoarthritis. Int J Biol Sci..

[23] Cai Z, Feng Y, Li C, Yang K, Sun T, Xu L (2018). Magnoflorine with hyaluronic acid gel promotes subchondral bone regeneration and attenuates cartilage degeneration in early osteoarthritis. Bone.

[24] Li C, Wu W, Jiao G, Chen Y, Liu HJRA (2018). Resveratrol attenuates inflammation and reduces matrix-metalloprotease expression by inducing autophagy via suppressing the Wnt/β-catenin signaling pathway in IL-1β-induced osteoarthritis chondrocytes. RSC advances..

[25] Jiang M, Li X, Yu X, Liu X, Xu X, He J (2017). Oral administration of resveratrol alleviates osteoarthritis pathology in C57BL/6J mice model induced by a high-fat diet. Mediators Inflamm.

[26] Shakibaei M, Csaki C, Nebrich S, Mobasheri A (2008). Resveratrol suppresses interleukin-1beta-induced inflammatory signaling and apoptosis in human articular chondrocytes: potential for use as a novel nutraceutical for the treatment of osteoarthritis. Biochem Pharmacol.

[27] Zhang Z, Leong DJ, Xu L, He Z, Wang A, Navati M (2016). Curcumin slows osteoarthritis progression and relieves osteoarthritis-associated pain symptoms in a post-traumatic osteoarthritis mouse model. Arthritis Res Ther..

[28] Shakibaei M, John T, Schulze-Tanzil G, Lehmann I, Mobasheri A (2007). Suppression of NF-kappaB activation by curcumin leads to inhibition of expression of cyclo-oxygenase-2 and matrix metalloproteinase-9 in human articular chondrocytes: implications for the treatment of osteoarthritis. Biochem Pharmacol.

[29] Feng Z, Zheng W, Li X, Lin J, Xie C, Li H (2017). Cryptotanshinone protects against IL-1beta-induced inflammation in human osteoarthritis chondrocytes and ameliorates the progression of osteoarthritis in mice. Int Immunopharmacol.

[30] Lu YC, Hsiao G, Lin KH, Hsieh MS, Jayakumar T, Wu TS (2013). Cinnamophilin isolated from *Cinnamomum philippinense* protects against collagen degradation in human chondrocytes. Phytother Res..

[31] Chen Y, Shou K, Gong C, Yang H, Yang Y, Bao T (2018). Anti-Inflammatory effect of geniposide on osteoarthritis by suppressing the activation of p38 MAPK signaling pathway. Biomed Res Int.

[32] Haseeb A, Ansari MY, Haqqi TM (2017). Harpagoside suppresses IL-6 expression in primary human osteoarthritis chondrocytes. J Orthop Res.

[33] Lin J, Li X, Qi W, Yan Y, Chen K, Xue X (2018). Isofraxidin inhibits interleukin-1beta induced inflammatory response in human osteoarthritis chondrocytes. Int Immunopharmacol.

[34] Jin J, Yu X, Hu Z, Tang S, Zhong X, Xu J (2018). Isofraxidin targets the TLR4/MD-2 axis to prevent osteoarthritis development. Food Funct..

[35] Fu D, Shang X, Ni Z, Shi G (2016). Shikonin inhibits inflammation and chondrocyte apoptosis by regulation of the PI3K/Akt signaling pathway in a rat model of osteoarthritis. Exp Ther Med..

[36] Jia T, Qiao J, Guan D, Chen T (2017). Anti-inflammatory effects of licochalcone A on IL-1beta-stimulated human osteoarthritis chondrocytes. Inflammation..

[37] Kang S, Siddiqi MH, Yoon SJ, Ahn S, Noh HY, Kumar NS (2016). Therapeutic potential of compound K as an IKK inhibitor with implications for osteoarthritis prevention: an in silico and in vitro study. Vitro Cell Dev Biol Anim..

[38] Liu Z, Lang Y, Li L, Liang Z, Deng Y, Fang R (2018). Effect of emodin on chondrocyte viability in an in vitro model of osteoarthritis. Exp Ther Med..

[39] Ding QH, Ye CY, Chen EM, Zhang W, Wang XH (2018). Emodin ameliorates cartilage degradation in osteoarthritis by inhibiting NF-kappaB and Wnt/beta-catenin signaling in vitro and in-vivo. Int Immunopharmacol.

[40] Zhang W, Li R, Wang S, Mu F, Jia P (2013). Effect of Chinese traditional herb *Epimedium grandiflorum C. Morren* and its extract Icariin on osteoarthritis via suppressing NF-kappaB pathway. Indian J Exp Biol.

[41] Zhang W, Li R, Wang S, Zhou X, Zhong Y (2011). Study of molecular mechanisms of fuyuan capsule, icariin and arasaponin R1 in treatment of osteoarthritis. Zhongguo Zhong Yao Za Zhi..

[42] Zhao Y, Li Z, Wang W, Zhang H, Chen J, Su P (2016). Naringin protects against cartilage destruction in osteoarthritis through repression of NF-kappaB signaling pathway. Inflammation..

[43] Ding Q, Zhong H, Qi Y, Cheng Y, Li W, Yan S (2013). Anti-arthritic effects of crocin in interleukin-1beta-treated articular chondrocytes and cartilage in a rabbit osteoarthritic model. Inflamm Res.

[44] Zhou K, Hu L, Liao W, Yin D, Rui F (2016). Coptisine prevented il-beta-induced expression of inflammatory mediators in chondrocytes. Inflammation..

[45] Ying X, Chen X, Cheng S, Shen Y, Peng L, Xu HZ (2013). Piperine inhibits IL-beta induced expression of inflammatory mediators in human osteoarthritis chondrocyte. Int Immunopharmacol.

[46] Zheng W, Zhang H, Jin Y, Wang Q, Chen L, Feng Z (2017). Butein inhibits IL-1beta-induced inflammatory response in human osteoarthritis chondrocytes and slows the progression of osteoarthritis in mice. Int Immunopharmacol.

[47] Zhuang Z, Ye G, Huang B (2017). Kaempferol alleviates the interleukin-1beta-induced inflammation in rat osteoarthritis chondrocytes via suppression of NF-kappaB. Med Sci Monit.

[48] Wang CL, Li, Wang CD, Xiao F, Zhu JF, Shen C (2017). Anti-inflammatory and anti-osteoarthritis effects of tectorigenin. Biol Open..

[49] Wang Z, Huang J, Zhou S, Luo F, Xu W, Wang Q (2017). Anemonin attenuates osteoarthritis progression through inhibiting the activation of IL-1beta/NF-kappaB pathway. J Cell Mol Med.

[50] Chen YJ, Tsai KS, Chan DC, Lan KC, Chen CF, Yang RS (2014). Honokiol, a low molecular weight natural product, prevents inflammatory response and cartilage matrix degradation in human osteoarthritis chondrocytes. J Orthop Res.

[51] Chen J, Gu YT, Xie JJ, Wu CC, Xuan J, Guo WJ (2018). Gastrodin reduces IL-1beta-induced apoptosis, inflammation, and matrix catabolism in osteoarthritis chondrocytes and attenuates rat cartilage degeneration in vivo. Biomed Pharmacother.

[52] Tang X, Yu F, Sun S, Jiang H, Gong Z, Xu J (2013). Effect of astragaloside IV on IL-1β expression of degenerative joint cartilage in human knee osteoarthritis. J Nanjing U TCM..

[53] Peng L, Xie Z, Pei J, Wang B, Gao Y, Qu Y (2019). Puerarin alters the function of monocytes/macrophages and exhibits chondroprotection in mice. Mol Med Rep..

[54] Ding QH, Cheng Y, Chen WP, Zhong HM, Wang XH (2013). Celastrol, an inhibitor of heat shock protein 90beta potently suppresses the expression of matrix metalloproteinases, inducible nitric oxide synthase and cyclooxygenase-2 in primary human osteoarthritic chondrocytes. Eur J Pharmacol.

[55] Chen MP, Yang SH, Chou CH, Yang KC, Wu CC, Cheng YH (2010). The chondroprotective effects of ferulic acid on hydrogen peroxide-stimulated chondrocytes: inhibition of hydrogen peroxide-induced pro-inflammatory cytokines and metalloproteinase gene expression at the mRNA level. Inflamm Res.

[56] Liu M, Zhong S, Kong R, Shao H, Wang C, Piao H (2017). Paeonol alleviates interleukin-1beta-induced inflammatory responses in chondrocytes during osteoarthritis. Biomed Pharmacother.

[57] Zhao C, Liu Q, Wang K (2017). Artesunate attenuates ACLT-induced osteoarthritis by suppressing osteoclastogenesis and aberrant angiogenesis. Biomed Pharmacother.

[58] Pan Z, Chen N, Zhang C, Li C, Huang X, Ye C (2011). The effects of genistein on the expression of NO and ultra-microstructure of osteoarthritis cartilage and chondrocyte in OA rats. J Trad Chin Orthopedics Traumatol..

[59] Zhou Y, Huang Q, Zhang T, Chen P (2017). Research on the protective effects of TFDG on IL-1β-induced inflammatory injury in rat chondrocytes in vitro. J Tea Sci..

[60] Tsai PW, Lee YH, Chen LG, Lee CJ, Wang CC (2018). In vitro and in vivo anti-osteoarthritis Effects of 2,3,5,4′-tetrahydroxystilbene-2-O-beta-d-glucoside from polygonum multiflorum. Molecules.

[61] Wang J, Gao JS, Chen JW, Li F, Tian J (2012). Effect of resveratrol on cartilage protection and apoptosis inhibition in experimental osteoarthritis of rabbit. Rheumatol Int.

[62] Liu S, Yang H, Hu B, Zhang M (2017). Sirt1 regulates apoptosis and extracellular matrix degradation in resveratrol-treated osteoarthritis chondrocytes via the Wnt/beta-catenin signaling pathways. Exp Ther Med..

[63] Wendling D, Abbas W, Godfrin-Valnet M, Guillot X, Khan KA, Cedoz JP (2013). Resveratrol, a sirtuin 1 activator, increases IL-6 production by peripheral blood mononuclear cells of patients with knee osteoarthritis. Clin Epigenet..

[64] Wang Y, Gao J, Chen J, Li F, Tian J, Xie X (2009). Effect of resveratrol on apoptosis and expression of bal-2 and bax protein in articular chondrocytes of experimental osteoarthritis model. Chin J Rheumatol..

[65] Li XS, Chen H, Zhen P, Li SS, Zhou SH, Tian Q (2016). JAK2/STAT3 signal pathway mediating curcumin in cartilage cell metabolism of osteoarthritis. Zhongguo Gu Shang..

[66] Zhang G, Cao J, Yang E, Liang B, Ding J, Liang J (2018). Curcumin improves age-related and surgically induced osteoarthritis by promoting autophagy in mice. Biosci Rep.

[67] Zhou Y, Liu SQ, Yu L, He B, Wu SH, Zhao Q (2015). Berberine prevents nitric oxide-induced rat chondrocyte apoptosis and cartilage degeneration in a rat osteoarthritis model via AMPK and p38 MAPK signaling. Apoptosis.

[68] Hu PF, Chen WP, Tang JL, Bao JP, Wu LD (2011). Protective effects of berberine in an experimental rat osteoarthritis model. Phytother Res..

[69] Zhao H, Zhang T, Xia C, Shi L, Wang S, Zheng X (2014). Berberine ameliorates cartilage degeneration in interleukin-1beta-stimulated rat chondrocytes and in a rat model of osteoarthritis via Akt signalling. J Cell Mol Med.

[70] Ju XD, Deng M, Ao YF, Yu CL, Wang JQ, Yu JK (2010). The protective effect of tetramethylpyrazine on cartilage explants and chondrocytes. J Ethnopharmacol.

[71] Qi W, Ganxiang H, Yanfen H, Tang Y, Song Y, Cai Q (2016). Study on Time-effect and Dose-effect of Paeonol on the Apoptosis of Knee Osteoarthritis Articular Chon-drocyte in Rabbits and the mRNA Expression of Its Related Protein Bcl-2 and Bax. Chin Pharm..

[72] Ju XD, Deng M, Ao YF, Yu CL, Wang JQ, Yu JK (2010). Protective effect of sinomenine on cartilage degradation and chondrocytes apoptosis. Yakugaku Zasshi.

[73] Liu J, Meng Q, Jing H, Zhou S (2017). Astragaloside IV protects against apoptosis in human degenerative chondrocytes through autophagy activation. Mol Med Rep..

[74] Zhang P (2017). GinsenosideRg5 treatment inhibits apoptosis of chondrocytes and degradation of cartilage matrix in a rat model of osteoarthritis. Oncol Rep.

[75] Zheng W, Lin P, Ma Y, Shao X, Chen H, Chen D (2017). Psoralen promotes the expression of cyclin D1 in chondrocytes via the Wnt/beta-catenin signaling pathway. Int J Mol Med.

[76] Wang W, Zeng L, Wang ZM, Zhang S, Rong XF, Li RH (2015). Ginsenoside Rb1 inhibits matrix metalloproteinase 13 through down-regulating Notch signaling pathway in osteoarthritis. Exp Biol Med (Maywood)..

[77] Li W, Cai L, Zhang Y, Cui L, Shen G (2015). Intra-articular resveratrol injection prevents osteoarthritis progression in a mouse model by activating SIRT1 and thereby silencing HIF-2alpha. J Orthop Res.

[78] Luo Y, Zhang Y, Huang Y (2018). Icariin reduces cartilage degeneration in a mouse model of osteoarthritis and is associated with the changes in expression of indian hedgehog and parathyroid hormone-related protein. Med Sci Monit.

[79] Zeng L, Rong XF, Li RH, Wu XY (2017). Icariin inhibits MMP1, MMP3 and MMP13 expression through MAPK pathways in IL1beta stimulated SW1353 chondrosarcoma cells. Mol Med Rep..

[80] Zhang X, Shi Y, Zhang Z, Yang Z, Huang G (2018). Intra-articular delivery of tetramethylpyrazine microspheres with enhanced articular cavity retention for treating osteoarthritis. Asian J Pharm Sci.

[81] Meng X, Huang G, Hui N, Jiang H, Chen Y, Cao L (2012). Effect of astragaloside IV on the expressions of MMP-1 and MMP-3 mRNA in chondrocytes of osteoarthritis patients. J Liaoning Univ Tradit Chin Med..

[82] Cui Z, Crane J, Xie H, Jin X, Zhen G, Li C (2016). Halofuginone attenuates osteoarthritis by inhibition of TGF-beta activity and H-type vessel formation in subchondral bone. Ann Rheum Dis.

[83] Liang Y, Chen S, Yang Y, Lan C, Zhang G, Ji Z (2016). Effect of puerarin on TIMP3, MMP-9 expression and methylation in chondrocytes of rat osteoarthritis. Int J Clin Exp Med.

[84] Qiu L, Luo Y, Chen X (2018). Quercetin attenuates mitochondrial dysfunction and biogenesis via upregulated AMPK/SIRT1 signaling pathway in OA rats. Biomed Pharmacother.

[85] Wang W, Ha C, Lin T, Wang D, Wang Y, Gong M (2018). Celastrol attenuates pain and cartilage damage via SDF-1/CXCR4 signalling pathway in osteoarthritis rats. J Pharm Pharmacol.

[86] Kim G, Kim H, Lee D, Choi S, Lee S, Noh HY (2013). Effects of supercritical fluid extract, shikonin and acetylshikonin from *Lithospermum erythrorhizon* on chondrocytes and MIA-induced osteoarthritis in rats. Korean J Med Crop Sci..

[87] Chen Y, Lin S, Sun Y, Pan X, Xiao L, Zou L (2016). Translational potential of ginsenoside Rb1 in managing progression of osteoarthritis. J Orthop Translat..

[88] Wang L, Shan H, Wang B, Wang N, Zhou Z, Pan C (2018). Puerarin attenuates osteoarthritis via upregulating AMP-activated protein kinase/proliferator-activated receptor-gamma coactivator-1 signaling pathway in osteoarthritis rats. Pharmacology.

[89] Xie P, Yu X, Chai S, Cao X, Sun H, Chen Q (2018). Ligustrazine for early-stage knee osteoarthritis in rats: changes in the expression levels of type II collagen fiber alpha 1, vascular endothelial growth factor and miR20b in the cartilage. Chin J Tissue Eng Res..

[90] Tang X, Yu F, Sun S, Jiang H, Gong Z, Wang Y (2013). Astragaloside IV preventing cartilage degeneration of patients with knee osteoarthritis by adjusting VEGF mRNA expression. J Liaoning Univ TCM..

[91] Mu W, Xu B, Ma H, Li J, Ji B, Zhang Z (2018). Halofuginone attenuates osteoarthritis by rescuing bone remodeling in subchondral bone through oral gavage. Front Pharmacol..

[92] Wang Z, Ding L, Zhang S, Jiang T, Yang Y, Li R (2014). Effects of icariin on the regulation of the OPG-RANKL-RANK system are mediated through the MAPK pathways in IL-1beta-stimulated human SW1353 chondrosarcoma cells. Int J Mol Med.

[93] Zhou B, Lu X, Tang Z, Liu D, Zhou Y, Zeng P (2017). Influence of sinomenine upon mesenchymal stem cells in osteoclastogenesis. Biomed Pharmacother.

[94] Abed É, Delalandre A, Pelletier J-P, Martel-Pelletier J, Lajeunesse D (2014). Resveratrol Regulates the Wnt/ß-catenin pathway in human osteoarthritis osteoblasts. Osteoarthr Cartilage..

[95] Pan L, Zhang Y, Chen N, Yang L (2017). Icariin regulates cellular functions and gene expression of osteoarthritis patient-derived human fibroblast-like synoviocytes. Int J Mol Sci.

[96] Zhang Y, Zeng Y (2019). Curcumin reduces inflammation in knee osteoarthritis rats through blocking TLR4/MyD88/NF-kappaB signal pathway. Drug Dev Res.

[97] Zeng JJ, Wang HD, Shen ZW, Yao XD, Wu CJ, Pan T (2019). Curcumin inhibits proliferation of synovial cells by downregulating expression of matrix metalloproteinase-3 in osteoarthritis. Orthop Surg..

[98] Wei B, Zhang Y, Tang L, Ji Y, Yan C, Zhang X (2019). Protective effects of quercetin against inflammation and oxidative stress in a rabbit model of knee osteoarthritis. Drug Dev Res.

[99] Yang HQ, Chen LR (2008). Effects of sinomenine on synovial fluid and serum content of interleukin-1beta in rabbits with osteoarthritis. Zhong Xi Yi Jie He Xue Bao..

[100] Bai Z, Guo XH, Tang C, Yue ST, Shi L, Qiang B (2018). Effects of artesunate on the expressions of insulin-like growth factor-1, osteopontin and C-telopeptides of type II collagen in a rat model of osteoarthritis. Pharmacology.

[101] Xiao J, Xu Y, Wang J, Feng J, Shi Z (2011). Bilateral knee lipoma arborescens combined with osteoarthritis in elderly patients. J Int Med Res.

[102] Zheng J, Wang R, Kou J (2016). Effects of Intra-articular injection of sinomenine on morphology, MMP-13 level and cartilage oligomeric matrix protein of rabbit knee osteoarthritis model. Chin J Inform TCM..

[103] Aaron R (2016). Circulatory pathology in osteoarthritis Skeletal Circulation in Clinical Practiceed.

[104] Permatasari DA, Karliana D, Iskandarsyah I, Arsianti A, Bahtiar A (2019). Quercetin prevent proteoglycan destruction by inhibits matrix metalloproteinase-9, matrix metalloproteinase-13, a disintegrin and metalloproteinase with thrombospondin motifs-5 expressions on osteoarthritis model rats. J Adv Pharm Technol Res..

[105] Marouf BH, Hussain SA, Ali ZS, Ahmmad RS (2018). Resveratrol supplementation reduces pain and inflammation in knee osteoarthritis patients treated with meloxicam: a randomized placebo-controlled study. J Med Food.

[106] Xu Q, Zhang ZF, Sun WX (2017). Effect of naringin on monosodium iodoacetate-induced osteoarthritis pain in rats. Med Sci Monit.

[107] Qian K, Zhang L, Shi K (2019). Triptolide prevents osteoarthritis via inhibiting hsa-miR-20b. Inflammopharmacology.

[108] Gao K, Wang S, Wang Q (2017). Effect of icariin on serum bone turnover markers expressions and histology changes in mouse osteoarthritis model. Zhongguo Xiu Fu Chong Jian Wai Ke Za Zhi..

[109] Tong M, Gao G, Xiang D, Gao J (2009). Effect of resveratrol on blood rheology of osteoarthritis in rabbits. Chin J Mod Appl Pharm.

[110] Lei M, Guo C, Hua L, Xue S, Yu D, Zhang C (2017). Crocin attenuates joint pain and muscle dysfunction in osteoarthritis rat. Inflammation..

[111] Pires DE, Blundell TL, Ascher DB (2015). pkCSM: predicting small-molecule pharmacokinetic and toxicity properties using graph-based signatures. J Med Chem.

[112] Li XZ, Zhang SN (2019). Herbal compounds for rheumatoid arthritis: literatures review and cheminformatics prediction. Phytother Res..

[113] Nixon R, Coury J, Shah S, Chahine N, Goldstein T, Collins M (2017). Evaluation of TRB-n0224, a chemically modified curcumin for the treatment of osteoarthritis. Osteoarthr Cartilage..

[114] Li XZ, Zhang SN, Yang XY (2017). Combination of cheminformatics and bioinformatics to explore the chemical basis of the rhizomes and aerial parts of *Dioscorea nipponica Makino*. J Pharm Pharmacol.

[115] Li XZ, Zhu KY, Zhang SN (2019). Exploration of biolabel: a new pattern for the study on the nature of Chinese materia medica. World Sci Tech Mod Trad Chin Med Mater Med..

[116] Li XZ, Huang HJ, Zhang SN, Liu Q, Wang YM (2020). Label-free quantitative proteomics positions the effects and mechanisms of Herba Lysimachiae on synovial diseases based on biolabelled research pattern. J Chromatogr B.

[117] Zhang A, Sun H, Yan G, Han Y, Zhao Q, Wang X (2019). Chinmedomics: a powerful approach integrating metabolomics with serum pharmacochemistry to evaluate the efficacy of traditional Chinese medicine. Engineering..

[118] Ru J, Li P, Wang J, Zhou W, Li B, Huang C (2014). TCMSP: a database of systems pharmacology for drug discovery from herbal medicines. J Cheminform..

