# Supplementary material for: Recent advance in treatment of osteoarthritis by bioactive components from herbal medicine
Source: Chin Med. 2020 Aug 1;15:80. doi: 10.1186/s13020-020-00363-5 (PMC7395386; doi:10.1186/s13020-020-00363-5)
Supplement: Supplementary file 1 — Additional file 1: Figure S1. Molecular structures of bioactive components isolated from herbal medicines treating OA. [file 13020_2020_363_MOESM1_ESM.docx]

**Terpenoids**

|  |  |  |  |
| --- | --- | --- | --- |
| 1. artesunate | 2. astragaloside IV | 3. celastrol | 4. compound K |

|  | |  | |  | |  |
| --- | --- | --- | --- | --- | --- | --- |
| 5. crocin | | 6. cryptotanshinone | | 7. geniposide | |  |
|  |  | |  | |  | |
| 8. ginsenoside Rb1 | 9. ginsenoside Rg5 | | 10. harpagoside | | 11. triptolide | |

**Flavonoids**

|  |  |  |  |
| --- | --- | --- | --- |
| 12. butein | 13. genistein | 14. icariin | 15. kaempferol |

|  |  |  |  |
| --- | --- | --- | --- |
| 16. licochalcone A | 17. naringin | 18. puerarin | 19. quercetin |

|  |  |  |  |
| --- | --- | --- | --- |
| 20. tectorigenin | 21. theaflavin-3,3′-digallate |  |  |

**Alkaloids**

|  |  |  |  |
| --- | --- | --- | --- |
| 22. berberine | 23. coptisine | 24. halofuginone | 25. magnoflorine |

|  |  |  |  |
| --- | --- | --- | --- |
| 26. piperine | 27. sinomenine | 28. tetramethylpyrazine |  |

**Phenols**

|  |  |  |  |
| --- | --- | --- | --- |
| 29. 2, 3, 5, 4′-tetrahydroxystilbene -2-O-β-d-glucoside | 30. curcumin | 31. ferulic acid | 32. gastrodin |

|  |  |  |  |
| --- | --- | --- | --- |
| 33. paeonol | 34. resveratrol |  |  |

**Quinones**

|  |  |  |  |
| --- | --- | --- | --- |
| 35. acetylshikonin | 36. shikonin | 37. emodin |  |

**Coumarins**

|  |  |  |  |
| --- | --- | --- | --- |
| 38. isofraxidin | 39. psoralen |  |  |

**Lignans**

|  |  |  |  |
| --- | --- | --- | --- |
| 40. cinnamophilin | 41. honokiol |  |  |

**Steroids**

|  |  |  |  |
| --- | --- | --- | --- |
| 42. arasaponin R1 |  |  |  |

**Furans**

|  |  |  |  |
| --- | --- | --- | --- |
| 43. anemonin |  |  |  |

**Additional Figure S1 Molecular structures of bioactive components isolated from herbal medicines treating OA.**
